# Psychological Stress as a Risk Factor for Cardiovascular Disease: A Case-Control Study

**DOI:** 10.7759/cureus.10757

**Published:** 2020-10-01

**Authors:** FNU Satyjeet, Sidra Naz, Vinesh Kumar, Norah H Aung, Kanwal Bansari, Sana Irfan, Amber Rizwan

**Affiliations:** 1 Internal Medicine, Chandka Medical College, Larkana, PAK; 2 Internal Medicine, University of Health Sciences, Lahore, PAK; 3 Internal Medicine, Ghulam Mohammad Mahar Medical College, Sukkur, PAK; 4 Health Sciences, Western Illinois University, Macomb, USA; 5 Internal Medicine, Jinnah Sindh Medical University, Karachi, PAK; 6 Family Medicine, Jinnah Post Graduate Medical Center, Karachi, PAK

**Keywords:** psychological stress, pakistan, cardiovascular disease, myocardial infarction

## Abstract

Introduction

Stress is a phenomenon elicited in response to certain triggers that may be external or internal. Stress has been identified as a risk factor for various diseases, including cardiovascular disease. In this study, we aim to find an association between psychological stress and cardiovascular disease in the local setting

Methods

This case-control study was conducted in a tertiary care hospital in Pakistan from June 2019 to December 2019. One hundred and seventeen (117) patients with myocardial infarction and unstable angina were enrolled in the case group. One hundred and ten (110) patient’s attendants were enrolled in the study as controlled.

Results

The risk of a cardiovascular event was higher in patients with a history of social isolation (OR, 2.47), marital stress (OR, 2.28), work stress (OR, 3.2), childhood abuse (OR, 2.78), or trauma (OR, 2.67).

Conclusion

Psychological stress is an important risk for cardiovascular disease, which is often overlooked. Efforts should be made to include questions related to psychological stress in the history-taking of patients at risk of a cardiovascular event and the management plan should include psychological counseling.

## Introduction

Stress is a phenomenon elicited in response to certain triggers that may be external or internal. In order to recompense, the human body counters with what is known as stress responses. Depending on certain characteristics of triggers, namely, duration, category, and intensity, the effects exerted upon the body can be as mild as homeostatic changes to as severe as lethal outcomes. Thus, in most diseases, stress is one of the major factors responsible for unfavorable outcomes and the pathophysiology of the disease, for instance, people obliged to work or live under tense circumstances are likely to develop many diseases in the long run [1].

The aftereffects of extreme and prolonged distress can lead to psychological trauma, which may lead to morbid outcomes [2]. One of the adverse outcomes of persistent stress is on cardiovascular health [3-4]. The above makes it equivalent to the primary risk factors associated with cardiovascular disease (CVD) [5-7]. Andrew Steptoe in his statistical analysis revealed the prevalence of CVD among socially apathetic populations, which is about 50% (pooled relative risk = 1.5, 95% CI: 1.2-1.9) [8]. However, a 40% population working in pressured environments is likely to develop CVD (pooled relative risk = 1.4, 95% CI: 1.2-1.8) [8].

Admittedly, global researches have been carried out to ascertain a link between CVD and stress but very limited data has been gathered among the regional population to conclude an association between both. In this study, we aim to find an association between psychological stress and cardiovascular disease in the local setting.

## Materials and methods

This observational case-control study was conducted in the cardiovascular unit of a tertiary care hospital in Pakistan from June 2019 to December 2019. One hundred and seventeen (117) patients with myocardial infarction and unstable angina, who presented to the hospital during the duration of the study, were enrolled in the case group. The diagnosis was made using electrocardiogram (ECG) findings and troponin levels. One hundred and ten (110) patient’s attendants were enrolled in the study as controlled. A written consent letter was taken. The study was approved by the institution review board.

History, including questions related to stress, and demographics were noted in a self-administrated questionnaire. The analysis was done with the Statistical Package for the Social Sciences (SPSS®) software (version 23.0; IBM Corp., Armonk, NY). Numerical data were presented as means ± standard deviations (SD). Categorical data were presented as frequencies and percentages. The data were compared using the t-test and chi-square, and the odds ratio was used as appropriate. A p-value of less than 0.05 indicated that the null hypothesis is not valid, and there is a significant difference between variables.

## Results

The mean age of participants in the case group was 48 ± 11 years and in the control group, it was 46 ± 10. The risk of cardiovascular events was higher in patients who had a history of social isolation, marital and work stress, childhood abuse, and past trauma (Table 1).

**Table 1:** Comparison of case and control groups CVD, cardiovascular disease; OR, odds ratio Legend: * Chi-square was used; ** independent t-test was used; *** odds ratio was calculated

Characteristics	Patients with CVD (Case group; 117)	Patients without CVD (Control group; 110)	P-value or OR ratio
Gender: Male; Female	68 (58.1%); 49 (41.9%)	59 (53.6%); 51 (46.4%)	0.46*
Mean + SD age in years	48 ± 11	46 ± 10	0.15**
Smoking	52 (44.4%)	41 (37.2%)	1.34***
Family history of CVD	21 (17.9%)	18 (16.3%)	1.11***
Physiological stress			
Economic stress	36 (30.7%)	28 (25.4%)	1.30***
Social isolation	19 (16.2%)	08 (7.3%)	2.47***
Marital stress	31 (26.4%)	15 (13.6%)	2.28***
Childhood abuse	21 (17.9%)	08 (7.3%)	2.78***
History of trauma	18 (15.3%)	07 (6.4%)	2.67***
Work stress	33 (28.2%)	12 (10.9%)	3.22***

## Discussion

In this study, a higher frequency of marital problems, child abuse, social isolation, history of some trauma, and work stress was present in patients with cardiovascular diseases. However, in this study, economic instability was not significant because most of the case and control participants belonged to the same families; hence, earning was similar between the groups.

The association of congenital heart defects (CHD) with chronic stress, both early life and adulthood, is as much as 40%-60% approximately [9]. Early life stressors, such as parental substance use, sexual abuse, parental separation, parental disease, or death, and chronic stressors like poor socioeconomic status are discussed by most of the evidence. However, adult stressors, such as the death of a child or spouse, marital problems, and care for a sick spouse at home, are linked to increased risk for CHD, even though the most studied stressors are social isolation and stress at work [8]. In a study in which community surveys in 10 countries were analyzed, the risk of heart disease, in those who reported three or more childhood problems, was more than twofold as compared to those who reported none [9]. There is reportedly an increased risk of hypertension, hyperlipidemia, obesity, and cardiovascular disease in patients with post-traumatic stress disorder [10]. The Stockholm female coronary risk study reports marital stress worsens the disease prognosis in females with CHD [11].

The interconnected networks of the brain, which involve complex functions like cognition and emotions, is considered to have an important role, even though there are many other factors involved in the processing of CVD. Amygdala has a key role [12]. During stress, it causes autonomic, hormonal, and behavioral changes associated with it [13]. The efferent projections of the amygdala to the brainstem assist in the sympathetic responses to stress [2]. In murine models, stress causes an increase in the proliferation of hematopoietic stem cells and progenitor cells in the bone marrow, hence accelerating the innate immune cell output and cytokine production, and reinforces atherosclerosis [14-18].

Stress causes increased activity of the amygdala such as in post-traumatic stress disorder, anxiety, and depression. Cellular glycolysis shown by F-fluorodeoxyglucose positron emission tomography/computed tomography (F-FDG PET/CT) can be used to simultaneously quantify regional brain activity [19]. A study conducted on patients from various medical centers reported that patients with post-traumatic stress disorder (PTSD) were at a higher risk to experience myocardial ischemia on exercise treadmill testing even after controlling other risk factors, including some psychological factors [20]. Studies report that this mental stress-induced ischemia is seldom associated with chest pain or other typical symptoms of ischemia but increases the risk of recurrent CVD events and hence higher mortality [20].

As per our knowledge, this is the first study in Pakistan that aims to find the association of psychological stress with CVD. However, there are certain limitations to this study. First, since it was a case-control study, the association could not be proved with absolute certainty. Second, as it was conducted in a single center, there may have been a decreased diversity in the sample. Along with the previous studies conducted, this study provides objective evidence of the effects of psychological stressors on cardiovascular health and suggests that cardiovascular events may be a result of a combination of atherosclerotic plaques and acute myocardial ischemia triggered by stress.

## Conclusions

We believe that certain triggers can favor the occurrence of cardiovascular diseases such as marital and child abuse, history of traumatic past events, and stressful work environment. It would likely be beneficial if attending physicians were to explore these factors when assessing patients for cardiovascular diseases. This will help in modifying patient treatment plans corresponding to the psychosocial factors contributing to CVD with the help of cognitive and behavioral therapies. Further, large-scale, multicenter prospective studies are needed to establish psychological stress as a risk factor for a cardiovascular event in the local setting.
